# Compared analysis with a high-quality genome of weedy rice reveals the evolutionary game of de-domestication

**DOI:** 10.3389/fpls.2022.1065449

**Published:** 2022-11-18

**Authors:** Jie Ma, Hua Wei, Xiaoman Yu, Yang Lv, Yu Zhang, Qian Qian, Lianguang Shang, Longbiao Guo

**Affiliations:** ^1^ State Key Lab for Rice Biology, China National Rice Research Institute, Chinese Academy of Agricultural Sciences, Hangzhou, China; ^2^ Shenzhen Branch, Guangdong Laboratory of Lingnan Modern Agriculture, Genome Analysis Laboratory of the Ministry of Agriculture and Rural Affairs, Agricultural Genomics Institute at Shenzhen, Chinese Academy of Agricultural Sciences, Shenzhen, Guangdong, China

**Keywords:** weedy rice, genome assembly, structural variation, pan-genome graph, de-domestication

## Abstract

The weedy rice (*Oryza sativa* f. *spontanea*) harbors large numbers of excellent traits and genetic diversities, which serves as a valuable germplasm resource and has been considered as a typical material for research about de-domestication. However, there are relatively few reference genomes on weedy rice that severely limit exploiting these genetic resources and revealing more details about de-domestication events. In this study, a high-quality genome (~376.4 Mb) of weedy rice A02 was assembled based on Nanopore ultra-long platform with a coverage depth of about 79.3× and 35,423 genes were predicted. Compared to Nipponbare genome, 5,574 structural variations (SVs) were found in A02. Based on super pan-genome graph, population SVs of 238 weedy rice and cultivated rice accessions were identified using public resequencing data. Furthermore, the de-domestication sites of weedy rice and domestication sites of wild rice were analyzed and compared based on SVs and single-nucleotide polymorphisms (SNPs). Interestingly, an average of 2,198 genes about de-domestication could only be found by *F*_ST_ analysis based on SVs (SV-*F*_ST_) while not by *F*_ST_ analysis based on SNPs (SNP-*F*_ST_) in divergent region. Additionally, there was a low overlap between domestication and de-domestication intervals, which demonstrated that two different mechanisms existed in these events. Our finding could facilitate pinpointing of the evolutionary events that had shaped the genomic architecture of wild, cultivated, and weedy rice, and provide a good foundation for cloning of the superior alleles for breeding.

## Introduction

Different from cultivated rice, weedy rice (*Oryza sativa* f. *spontanea*) possesses stronger seed dormancy, reproductive ability, and higher phenotypic plasticity. Similar with wild rice (*Oryza rufipogon*), weedy rice also has features such as a red pericarp, a black hull, and seed shattering. Furthermore, weedy rice has stronger tolerance to biotic and abiotic stress than cultivated rice. On the whole, weedy rice is a gold mine, which contains numerous useful genetic resources for rice functional genomic studies and breeding (Wu et al., 2022). Since weedy rice has these reproductive advantages, many quantitative trait locus (QTLs) mapping traits for seed shattering and dormancy have been identified during decades past, such as shattering loci of *sh4* and *qSH1* (Qi et al., 2015) and seed dormancy and red pericarp major loci of *qSD7-1*/*qPC7* (Gu et al., 2004; Gu et al., 2011). Pan-genome of rice not only presents more useful genetic information for molecular breeding by design but also provides insights into the evolutionary events (Shang et al., 2022). The pan-genome of cultivated rice has been constructed, while weedy rice was missed in the currently available rice pan-genome (Wang et al., 2018; Zhao et al., 2018; Qin et al., 2021; Zhang et al., 2022). Therefore, few clues were provided to further understand the molecular mechanism of cultivated rice de-domestication.

De-domestication is an interesting phenomenon in both plants and animal, which denotes that the domesticated crops and livestock reacquire components of wild like traits to form independent reproducing population (Ellstrand et al., 2010; Gering et al., 2019). If de-domestication constantly appears in crop fields, it usually leads to weeds, such as weedy rice (Qiu et al., 2020), weedy barely (Zeng et al., 2018) and weedy sunflower (Presotto et al., 2011). In rice, multiple studies identified that many loci (*sh4*, *qSH1*, *SSH1*, *OSH15* and *GRF4*) were involved in shaping the loss of seed shattering, among which *sh4* and *qSH1* were the major loci (Li et al., 2006; Sun et al., 2016; Yoon et al., 2017; Jiang et al., 2019). For example, the cultivated rice contains the G-to-T mutation of *sh4* underlying the loss of seed shattering. Under natural selection, de-domestication could provide valuable genetic resources for crop breeding. To elucidate the mysterious of de-domestication will expand our understanding of the evolutionary process of crops.

Previous studies showed that an increasing number of de-domestication events have been revealed in crops, such as *Oryza sativa* (Li et al., 2017; Qiu et al., 2017; Sun et al., 2019; Qiu et al., 2020)*, Hordeum agriocrithon* (Zeng et al., 2018), *Triticum aestivum* (Guo et al., 2020), *Sorghum bicolor* (Morrell et al., 2005), *Secale cereale* (Morrell et al., 2005), *Olea europaea* (Mekuria et al., 2002) and so on. However, of these events only three have been confirmed by genomic studies including rice, barley, and wheat. Therefore, the large scale and scope of genomic studies in crops will be helpful for confirming the de-domestication events and revealing more details about this event.

High-quality pan-genome can represent the full genetic information of a population and provide a new foundation for exploitation of genetic resources for crop improvement, which has been used to deeply analyze the large number of genetic variations, functional genes, species origin and domestication in rice (Wang et al., 2018; Zhao et al., 2018; Liu et al., 2020). Structural variation (SV), presence/absence variation (PAV) and gene copy number variation (gCNV) are the main causes of individual genomic differences and the core of pan-genome research. Among them, SV plays crucial role in crop evolution, domestication, and improvement. For example, Kou et al. (2020) used SV to study domestication by contrasting between Asian rice (*Oryza sativa*) and its wild relative *O. rufipogon* and found that SVs contributed to the domestication in rice. Recent studies also revealed the diverse genetic mechanism of rice de-domestication through the whole-genome sequencing of 524 global weedy rice and comparative analysis with accordingly cultivated rice. They mainly conducted single nucleotide polymorphism (SNP) across 1,003 samples genomic analysis (Qiu et al., 2020). However, the function of SV in de-domestication of rice has yet to be verified. Additionally, crop-weed genome comparison can provide new insights on the genetic mechanism of cultivated rice de-domestication process. In this study, Nanopore sequencing and assembly were performed on *japonica* weedy rice A02 and obtained a high-quality genetic map. Additionally, we used a variety of data source including super pan-genome graph to detect SVs in cultivated and weedy rice and evaluated the function and accuracy of SV which was performed as a tool to study de-domestication for weedy rice. And the de-domestication results were used to compare with domestication events of ordinary wild rice and cultivated rice populations.

## Materials and methods

### Plant materials and data preparation

High-latitude weedy rice A02 from Liaoning province was selected in our research and was grown in the greenhouse of China National Rice Research Institute. The Illumina resequencing data of 238 weedy rice and cultivated rice accessions used in this part were derived from public data (Qiu et al., 2017), which include 155 weedy rice samples from four representative provinces of Liaoning (LN), Ningxia (NX), Jiangsu (JS) and Guangdong (GD) in China, 76 local cultivated rice and 7 weedy rice accessions from the United States and South Korea. And the Illumina sequencing data of 26 Asian wild rice samples were collected from Shang et al. (2022). The super pan-genome involved in structural variation detection was constructed by Nanopore sequencing data of rice germplasm materials (Shang et al., 2022) based on Nipponbare reference genome (MSUv7) (Kawahara et al., 2013).

### Illumina sequencing

gDNA of A02 for short-read length sequencing was extracted from leaves of two-week-old seedlings with the CTAB method. The index libraries were constructed by using the New England Biolabs (NEB) Next^®^ Ultra™ DNA Library Prep Kit for Illumina (NEB, Ipswich, MA, USA) according to the manufacturer’s instructions. After quality assessment, at least 0.2 µg gDNA was randomly fragmented by sonication. After size grading by electrophoresis, approximately 350 bp DNA fragments were purified with the AMPure XP system (Beckman Coulter, Beverly, USA) for library construction. It was then sequenced on the Xten platform (Illumina, San Diego, CA, USA).

### Nanopore ultra-long sequencing

For the ultra-long Nanopore library, approximately 8-10 µg DNA (>50 Kb) of A02 was selected using the SageHLS HMW library system (Sage Science, USA) and processed using Ligation Sequencing Kit 1D (SQK-LSK109, Oxford Nanopore Technologies, UK). About 800 ng DNA library was constructed and sequenced on PromethION (Oxford Nanopore) to obtain the original sequencing data following the manufacturer’s instructions.

### Transcriptome sequencing

The RNA of A02 was extracted from young leaves of one-week-old seedlings with a TRIzol kit. The index library was constructed by TruSeq RNA Library Preparation Kit (Illumina, USA). And RNA was sequenced using Illumina sequencing platform NovaSeq 6000.

### *De novo* genome assembly and evaluation

Based on ultra-long Nanopore sequencing platform, the qualified Nanopore ultra-long reads (NULRs) (quality value >7) of the weedy rice A02 were obtained. Then NULRs were assembled by NextDenovo v2.4 (https://github.com/Nextomics/NextDenovo). After preliminary assembly, the genome sequences were further corrected three times with 22.8 Gb (60.8×) of Illumina paired-end reads and the NULRs using NextPolish v1.0 (https://github.com/Nextomics/NextPolish). BUSCO v9 (Simão et al., 2015) was used to evaluate the assembled sequences, which means using single copy Embryophyta genes in a specific library to predict the genes status of existing sequences in the genome.

### Chromosome assembly

Ragtag v2.1.0 (https://github.com/malonge/RagTag) was used to link the contigs into the closet chromosomes according to the coverage of contigs with Nipponbare genome (Kawahara et al., 2013) as reference and the chromosome-level assemblies were generated.

### Gene annotation

For assembled A02 genome, the RepeatMasker v4.0.7 (www.repeatmasker.org) was used to mask the repetitive sequences. And the coding regions in the repeat-masked genome were predicted using Augustus v3.0.3 (Keller et al., 2011), SNAP v2006-07-28 (Leskovec and Sosic, 2016) and Fgenesh (http://www.softberry.com/). Proteins from four plant genomes (*Arabidopsis thaliana*, *Brachypodium distachyon*, *Os* and *Sorghum bicolor*) were downloaded from Phytozome (https://phytozome-next.jgi.doe.gov). These protein sequences were aligned to the assembly using tBLASTN v2.9.0+ (Camacho et al., 2009) with an *E*-value cutoff of 1e-5. Genewise v2.4.2 (Birney et al., 2004) was used to refine the alignment with parameters ‘-gff -quiet -silent -sum’. Raw RNA-seq reads were qualified by Trimmomatic v0.36 (Bolger et al., 2014) with parameters ‘ILLUMINACLIP : TruSeq3-PE.fa:2:30:10 LEADING:3 TRAILING:3 SLIDINGWINDOW:4:15 MINLEN:36’. The clean RNA-seq data were mapped to the assembly using HISAT2 v2.1.0 (Kim et al., 2019). StringTie2 v2.1.4 (Kovaka et al., 2019) was used to assemble the transcripts into gene models. All gene models were integrated by EvidenceModeler v1.1.1 (Haas et al., 2008) to obtain the gene annotation results.

### Transposable element annotation

The whole-genome TEs were annotated with the TE library in the Extensive *de novo* TE Annotator (EDTA, v1.9.6) (Ou et al., 2022) package.

### SNP and SV calling

Raw DNA-seq reads from A02 gDNA sample were trimmed by Trimmomatic (Bolger et al., 2014) with parameters ‘ILLUMINACLIP:2:30:10 MINLEN:75 LEADING:20 TRAILING:20 SLIDINGWINDOW:5:20’. Clean reads were mapped to the Nipponbare reference genome (Kawahara et al., 2013) by Burrows-Wheeler Aligner (BWA, v0.7.17-r1188) (Li and Durbin, 2009). SAMtools v1.8 (Danecek et al., 2021) and BCFtools v1.8 (Danecek et al., 2021) were used to call and filter (DP < 3 and quality score < 30) SNP.

To call SVs, NULRs of A02 were aligned to the Nipponbare genome (Kawahara et al., 2013) using minimap2 v2.17-r974-dirty (Li, 2018) and NGMLR v0.2.7 (Sedlazeck et al., 2018). Sniffles v1.0.11 (Sedlazeck et al., 2018) was used to call SVs with parameters ‘-I 50 -genotype’.

SV calling based on Illumina sequencing data: raw Illumina resequencing data of 238 accessions from public data (Qiu et al., 2017) were first trimmed by Trimmomatic (Bolger et al., 2014) with parameters ‘ILLUMINACLIP:2:40:15 MINLEN:100 LEADING:30 TRAILING:30 SLIDINGWINDOW:4:15’. After quality control, the clean reads were mapped to the super pan-genome (Shang et al., 2022) to call SV with the short reads comparison tools Giraffe (Siren et al., 2021) in vg toolkit v1.38.0 (Hickey et al., 2020). SURVIVOR v1.0.7 (Jeffares et al., 2017) was used to merge SVs called by each accession into a population genotype.

### Genetic differentiation (*F*_ST_) analysis

In the study of weedy rice genome variation, Qiu et al. (2017) divided weedy rice and local cultivated rice in China into four group: Guangdong (GD), Jiangsu (JS), Liaoning (LN) and Ningxia (NX) according to the geographical location. In order to avoid the noise effect caused by population structure mixing, we selected four subgroups (NX1, LN1, JS1 and GD1) with clear population structure published by Qiu et al. (2017) to analyze the genetic differentiation of weedy rice and cultivated rice. We performed *F*_ST_ analysis based on SVs (SV-*F*_ST_) and SNPs (SNP-*F*_ST_), respectively. VCFtools v0.1.13 (Danecek et al., 2011) was used to calculate SV-*F*_ST_ and SNP-*F*_ST_ between weedy rice and local cultivated rice in four regions, and between Asian wild rice and cultivated rice as well. SNP-*F*_ST_ was performed in a 100 Kb window size with a step size of 10 Kb, and the parameters of SV-*F*_ST_, referring to Kou et al. (2020), were set in a 20 Kb window size with a step size of 10 Kb. Through the above SNP-*F*_ST_ and SV-*F*_ST_, the windows with top 5% *F*_ST_ values were identified as highly differentiated intervals. Genes in these series of intervals were then annotated based on the Nipponbare reference genome (Kawahara et al., 2013).

## Results

### Assembly and validation of high-quality genome sequences of weedy rice

**Figure 1:**
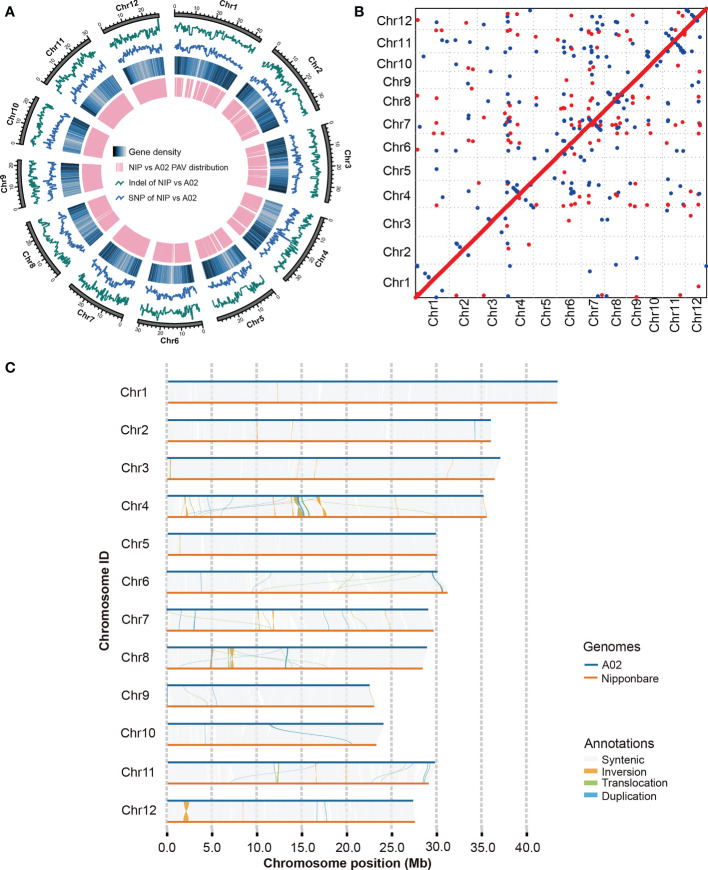
Assembly and analysis of A02 genome. **(A)** Landscape of A02 reference genome. Tracks from the outer to inner circles indicate: 1, indels between A02 and the Nipponbare reference genome; 2, SNPs in A02 with respect to Nipponbare; 3, gene density; 4, highlights of the PAV distribution between A02 and Nipponbare. NIP refers to Nipponbare. **(B)** The collinearity relationship between A02 genome and Nipponbare. The X-axis represents the genome of Nipponbare, and the Y-axis indicates the genome of weedy rice A02. **(C)** The collinearity of weedy rice A02 and Nipponbare.

To develop assemblies, 29.9 Gb (~79.3× coverage) of NULRs were generated for weedy rice A02 using Nanopore ultra-long platform in which the N50 and the longest reads can reach 52.9 Kb and 520.6 Kb respectively, of which the average read lengths are 30.7 Kb (**Supplementary Figure S1** and **Supplementary Table S1**). To improve accuracy, we used Illumina sequencing data to reduce the single-base and InDel error rate. After quality control of the Illumina reads data was completed, weedy rice retained 22.8 Gb of data with a coverage depth of about 60.8×. The Q20 of the data reached more than 97.0%. The assembled weedy rice genome was 376.4 Mb based on NULRs and contain 19 contigs, of which the contig N50 length was 29.39 Mb (**Table 1**). The 376.4 Mb sequences of A02 were anchored to 12 chromosomes, in which the BUSCO value reached more than 98% (**Figure 1A** and **Table 1**). The above data suggested that a high-quality genome of weedy rice was achieved.

**Table 1:** The statistics of Nanopore ultra-long reads assembly results of A02.

Sequencing type	NULRs
Total size of assembled genome (Mb)	376.4
Number of contigs	19
Largest contig (Mb)	43.54
Contig N50	29.39
Single base calling error (%)	0.02
BUSCOs (%)	98.00

### Annotation and comparison of the genome sequences for weedy rice

To determine the content of TEs, genome-wide annotation of the transposon sequence about weedy rice A02 was performed based on the TE library in the extensive *de novo* TE Annotator (Ou et al., 2022) and 183.9 Mb of TEs were identified, accounting for 48.95% of the entire genome. The most abundant class of TEs were the long terminal repeat (LTR), accounting for 22.75% of the genome (**Table 2**). Additionally, 35,423 genes were predicted in the weedy rice A02 genome, with an average gene length of 2.85 Kb. Subsequently, the genome sequence of the weedy rice at the chromosome level was aligned with the Nipponbare sequence using MUMmer 4.0.0 (Marçais et al., 2018), which suggested that the weedy rice had good collinearity compared with Nipponbare (**Figure 1B**). To better compare the variation of weedy rice A02, the genome-wide collinearity analysis of weedy rice A02 was carried out with the reference of Nipponbare genome sequence, and the results could show the SVs of A02 relative to Nipponbare (**Figure 1C**), which would provide a well-grounded basis for resolving the mechanisms associated with phenotypic variation in weedy rice.

**Table 2:** The statistics of transposable elements annotation results in A02.

Type of TEs		Number	Length (bp)	Proportion (%)
LTR	*copia*	11,521	13,390,207	3.56
	*gypsy*	46,404	70,063,097	18.65
	TRIM	2,887	577,556	0.15
	Unknown	1,377	1,450,919	0.39
TIR	CACTA	18,813	15,024,542	4.00
	*Mutator*	53,486	21,063,723	5.61
	*PIF/Harbinger*	43,381	9,896,554	2.63
	Tc1/*mariner*	54,224	16,098,042	4.29
	*h*AT	16,315	5,568,481	1.48
	Unknown	6,610	2,068,969	0.55
nonLTR	LINEs	6,968	3,861,853	1.03
	SINEs	6,420	1,228,455	0.33
	Unknown	302	544,273	0.14
nonTIR	*Helitron*	48,357	23,077,004	6.14
Total		317,065	183,913,675	48.95

### SV identification and analysis of de-domestication based on super pan-genome

**Figure 2:**
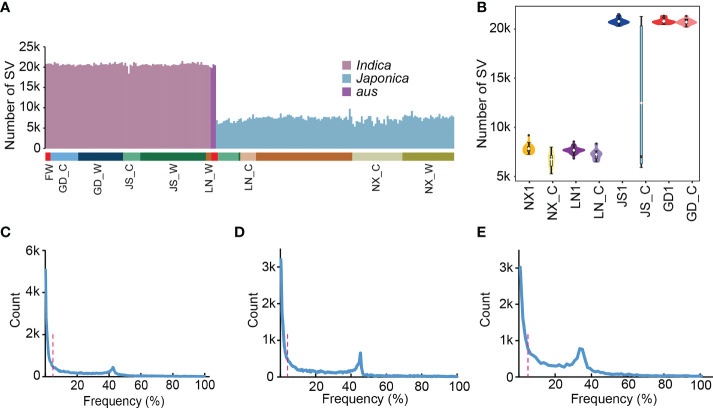
The statistics of SVs’ number and frequency. **(A)** The statistics of SVs’ number in 238 rice materials, which contains 162 weedy and 76 locally cultivated rice. LN for Liaoning, NX for Ningxia, JS for Jiangsu, GD for Guangdong and FW for weedy rice sampled outside of China. **(B)** The number of SVs in the four weedy rice subpopulations (NX1, LN1, JS1 and GD1) and the corresponding local cultivated rice. **(C-E)** Frequency of SVs in 238 rice materials **(C)**, 162 weedy rice **(D)**, and 76 local cultivated rice **(E)**. The red dashed line represents a frequency of 5%.

A super pan-genomic landscape of rice revealed extensive SVs, which enabled the accurate identification and characterization of their inter- and intraspecific diversity (Shang et al., 2022). Previous studies have showed that SVs underlie important crop improvement and domestication traits. To explore the role of SVs in de-domestication process, we calculated the SVs distribution of all weedy and cultivated rice genome based on super pan-genome graph. The results showed that the number of SVs was significantly different among materials, which was mainly related to the distance of evolutionary relationship between these materials and Nipponbare. For example, *indica* and *aus* subgroups generally contained more SVs than *japonica* subgroup with an average number of 20,327, while the *japonica* had an average of only 7,397 (**Figure 2A**). Next, we used the published data to count SV numbers for weedy rice subpopulations (NX1, LN1, JS1, and GD1) and the well-defined local cultivated rice (NX_C, LN_C, JS_C, and GD_C), respectively, which have been reported by Qiu et al. (2017). These data showed that the distribution of SVs in most groups was relatively uniform, except for JS_C (**Figure 2B**), which contains both *japonica* and *indica* materials. Additionally, we performed SV frequency statistics in 162 weedy and 76 cultivated rice and found that an average of 13.8% SVs only appeared in a single material and existed at private frequencies (**Figures 2C–E**), which further illustrated that SVs were generally harmful for plant growth and development.

**Figure 3:**
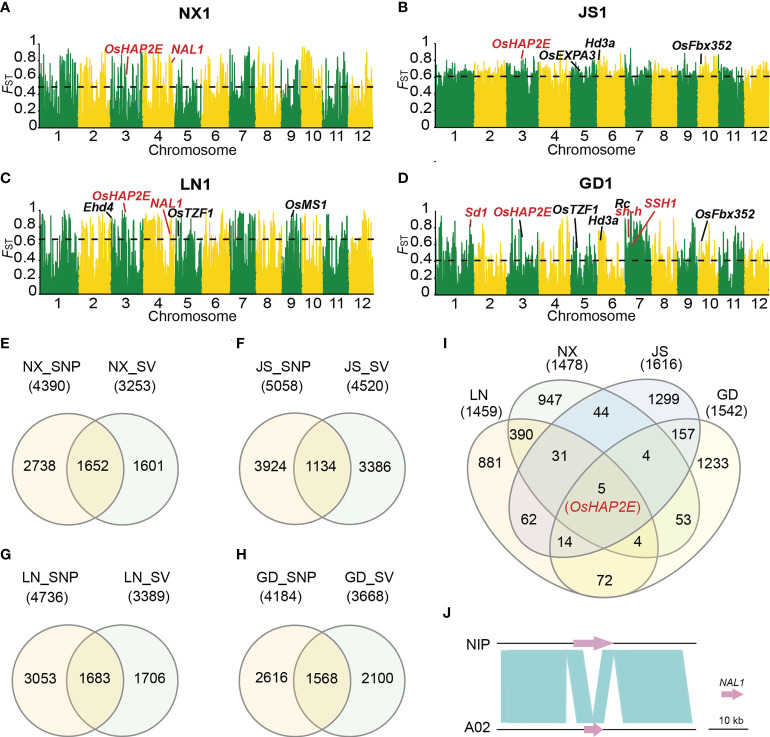
Genomic differentiation between weedy and cultivated rice from different locations. **(A-D)** SV-*F*_ST_ between weedy rice NX1 **(A)**, JS1 **(B)**, LN1 **(C)**, GD1 **(D)** and their corresponding cultivated rice. Genes emphasized in the text were marked in red. Horizontal dashed lines correspond to the top 5% threshold. **(E-H)** The intersection of divergent gene regions identified through SV-*F*_ST_ and SNP-*F*_ST_ in NX **(E)**, JS **(F)**, LN **(G)**, GD **(H)**. **(I)** The intersection of divergent windows identified in four regions through SV-*F*_ST_. **(J)** The variation of the *NAL1* gene between Nipponbare and A02. Most weedy rice lost a 5755 bp fragment in the region of the *NAL1* gene compared to Nipponbare.

To analyze the role of SVs in the process of rice de-domestication, we selected four subgroups with clear population structure (NX1, LN1, JS1 and GD1) to study population genetic differentiation. Through the SV-*F*_ST_ analysis of weedy and local cultivated rice populations, the highly differentiated ranges with top 5% SV-*F*_ST_ values were annotated in combination with the Nipponbare reference genome (Kawahara et al., 2013), among which these genes were mostly associated with heading date, stress or plant growth and development (**Figures 3A–D**). Subsequently, we used SNPs data that published by Qiu et al. (2017) to conduct SNP-*F*_ST_ analysis, and screened out highly differentiated windows in the same way. Then, the similarities and differences of differentiation intervals based on SNP and SV detection were further analyzed (**Figures 3E–H**). These data showed that the overlapped gene accounted for about 50% genes detected through SV-*F*_ST_ in four different areas (**Figures 3E–H**), indicating that SV-*F*_ST_ could verify the differentiation range obtained by SNP-*F*_ST_. Additionally, SV-*F*_ST_ also detected many highly differentiated genes that could not be found by SNP-*F*_ST_. To explore whether there are some common de-domestications related intervals in the four different regions, we investigated the simultaneous intersection of the highly differentiated intervals detected by SV-*F*_ST_ analysis and found a few identical windows (only five common genes) in different regional groups (**Figure 3I**). This indicated that different genes were selected to cope with the selection pressure and adapt to the local environment in the de-domestication process of weedy rice. Among them, the *OsHAP2E* gene was detected in all four regions (**Figure 3I**), which was reported to confer salt and drought tolerance, phytophthora ramorum and phytophthora albicanan resistance, improve the capacity of photosynthetic, and increase tiller number in rice (Alam et al., 2015). These characteristics were closely related to weedy rice, suggesting *OsHAP2E* might be involved in the de-domestication process to some extent. Moreover, a highly differentiated SV locus was identified, which was a 5,755 bp PAV, and a functional gene *NAL1* (*narrow leaf 1*) was detected in this genomic region in Nipponbare but not in A02 (**Figure 3J**). Previous studies have shown that *NAL1* positively regulate plant leaf width and plant height (Xu et al., 2015). Interestingly, this fragment was present in most of the cultivated rice while absent in weedy rice, indicating that it might be related to the characteristics of weedy rice. For example, in NX, this fragment was missed in 21 of 22 weedy rice, while only absent in one accession of the cultivated rice. Similar results were obtained in LN, where the PAV was absent in 38 of 40 weedy rice but present in all cultivated rice, which indicated a high degree of differentiation between weedy and cultivated rice.

### Comparison of domestication and de-domestication events

**Figure 4:**
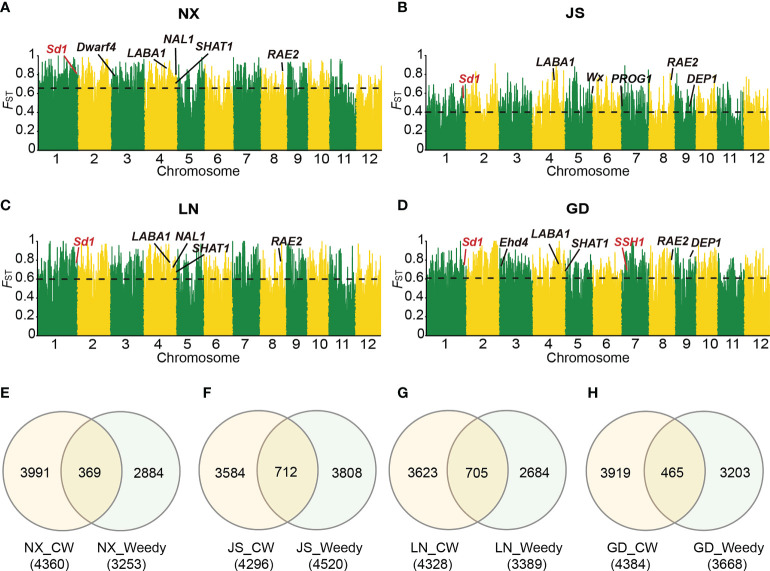
Genomic differentiation between Asian wild rice and cultivated rice from different locations. **(A-D)** SV-*F*_ST_ between Asian wild rice and cultivated rice from NX **(A)**, JS **(B)**, LN **(C)** and GD **(D)**, respectively. Genes emphasized in the text were marked in red. Horizontal dashed lines correspond to the top 5% threshold. **(E-H)** The intersection of divergent gene regions identified through SV-*F*_ST_ between Asian wild rice and cultivated rice and that between weedy and cultivated rice in four regions NX **(E)**, JS **(F)**, LN **(G)** and GD **(H)**, respectively. CW means cultivated rice.

To explore the similarities and differences between the mechanisms of the domestication and de-domestication processes, we further performed SV-*F*_ST_ analysis on 26 wild rice and cultivated rice in NX, LN, JS and GD to obtain the domestication related sites, respectively (**Figures 4A–D**). Subsequently, we compared these intervals between domestication and de-domestication, which showed these genes had a low overlap between domestication and de-domestication intervals in highly differentiated regions (**Figures 4E–H**). Unusually, these intervals had a high overlap that accounted for 20.8% of total (705/3389) in the LN area (**Figure 4G**). Thus, the mechanisms of domestication and de-domestication had some similarities. For example, *SSH1* (*suppression of shattering 1*), controlling shattering and grain size, was present in both domestication and de-domestication process detected by SV-*F*_ST_ in GD (**Figure 4D**). In the de-domestication region, the *sd1* (*Semi-dwarf* 1) gene was only detected in GD (**Figure 3D**), while *sd1* played an important role in domestication in four areas (**Figures 4A–D**). And large numbers of genes were only found in significantly differentiated regions related to the domestication process, such as *SHAT1* (*Shattering abortion1*) and *DEP1* (*Dense panicle1*), which could not be detected in de-domestication region (**Figures 3A–D**). On the contrary, shattering gene *sh-h* was only detected in the de-domestication related region (**Figure 3D**). Taken together, the de-domestication process from cultivated to weedy rice might have different genetic mechanisms with the domestication process from wild to cultivated rice. Our findings highlighted the underexplored role of SVs in de-domestication process and their widespread importance and utility in crop improvement.

## Discussion

Weedy rice (*Oryza sativa* f. *spontanea*) has many excellent traits, such as strong growth potential, environmental adaptability and multiple stresses resistance. High-quality genome sequences are the bases for mining functional genes, evolutionary origins, and genetic breeding. Here, we assembled a high-quality reference genome sequences of weedy rice A02 using Nanopore ultra-long platform. Compared with next-generation sequencing, Nanopore sequencing has greatly improved the read length, but there are still many assembly errors in long repetitive regions. Nanopore ultra-long sequencing is one of the best methods to solve this problem, which makes it possible to telomere-to-telomere assembly genome rapidly (Miga et al., 2020). NULRs have longer N50 and can effectively span large repeats in the genome, even the centromeric regions. The combination of these two sequencing platforms facilitated the assembly of the A02 high-quality genome, which had good collinearity with the reference genome of Nipponbare (**Figure 1B**). Through transposon annotation, we found that the weedy rice A02 contained 183.9 Mb transposons, accounting for 48.95% of the whole genome, and 35,423 protein-coding genes were predicted. Our study therefore provided genes pools for gene excavation to underlie the research about de-domestication of weedy rice.

Except for SNPs, SVs are the major source of genetic variation and tend to have a great impact on gene expression and phenotype in plants (Alonge et al., 2020). Consequently, using super pan-genome as a reference genome, SV-based and SNP-based genetic differentiation analysis were performed on 162 weedy rice populations mainly from GD, JS, LN and NX and 76 local cultivated rice populations, and found that SVs existed mainly at low frequencies in weedy rice populations compared with SNPs (**Figure 3**). By further analyzing the de-domestication loci in the four regions, we found that many loci could be detected through SV-*F*_ST_ but not SNP-*F*_ST_, and most of these de-domestication related SV loci were unique to each region. Additionally, we compared SV loci involved in the de-domestication and domestication events, and found that the shared differentiation loci only accounted for 20% (**Figure 4**), suggesting that the mechanism of de-domestication process of weedy rice might be different from that of the domestication process.

For another, SV-*F*_ST_ can detect many highly differentiated genes of weedy rice while the locally cultivated rice that cannot be found by SNP-*F*_ST_. For example, the *SSH1* can be specifically detected in the highly differentiated interval of weedy rice and cultivated rice in GD with SV-*F*_ST_. Previous studies have showed that *SSH1* controls the grain size and seed shattering by positively regulating the expression of two rice *REPLUMLESS* orthologs *qSH1* and *SH5*, which indicated that *SSH1* is valuable for improving rice seed shattering and grain yield (Jiang et al., 2019). Noticeably, a highly differentiated SV site, PAV of 5,755 bp, was present in most cultivated rice while lost in weedy rice, which was present on the functional gene *NAL1* that regulate leaf width and plant height (Xu et al., 2015) (**Figure 3J**). Genomic loci present in the highly differentiated regions might be crucial for the origin and adaption of weedy rice. At present, the excavation of genes controlling excellent traits such as stress resistance in weedy rice is still insufficient. Our research provided a solid foundation to further disclosing the genetic variation mechanisms of weedy rice and the cloning of beneficial genes for breeding.

## Conclusions

Herein, we assembled a high-quality weedy rice genome of A02 using Nanopore ultra-long platform, which provided a good foundation for understanding the molecular mechanism of cultivated rice de-domestication process and cloning of the superior alleles for breeding. Based on the super pan-genome and comparative genomic analysis, we found that two different mechanisms existed in domestication and de-domestication events, and different genes were selected to cope with the selection pressure and adapt to the local environment in the de-domestication process.

## Data availability statement

The datasets presented in this study can be found in online repositories. The names ofthe repository/repositories and accession number(s) can be found below: https://ngdc.cncb.ac.cn/gsub/, PRJCA011659. The SV information between the A02 genome and Nipponbare genome and the data of the SV genotyping information based on the super pan-genome are available in https://zenodo.org/record/7265880#.Y187b-RBxD9.

## Author contributions

LG, LS, and QQ designed the experiment. JM, XY, YL, and YZ performed analysis and interpretation of the data. JM and HW drafted the manuscript. All authors contributed to the article and approved the submitted version.

## Funding

This work was supported by grants from The National Natural Science Foundation of China (Grant No.32101718 and No. 3221101587); Hainan Yazhou Bay Seed Laboratory Project (B21HJ0223).

## Conflict of interest

The authors declare that the research was conducted in the absence of any commercial or financial relationships that could be construed as a potential conflict of interest.

## Publisher’s note

All claims expressed in this article are solely those of the authors and do not necessarily represent those of their affiliated organizations, or those of the publisher, the editors and the reviewers. Any product that may be evaluated in this article, or claim that may be made by its manufacturer, is not guaranteed or endorsed by the publisher.
