# Supplementary material for: Compared analysis with a high-quality genome of weedy rice reveals the evolutionary game of de-domestication
Source: Front Plant Sci. 2022 Nov 18;13:1065449. doi: 10.3389/fpls.2022.1065449 (PMC9716140; doi:10.3389/fpls.2022.1065449)
Supplement: Supplementary file 1 [file DataSheet_1.docx]

***Supplementary Material***

**Supplementary Figure S1**| The Nanopore ultra-long reads distribution of weedy rice A02.


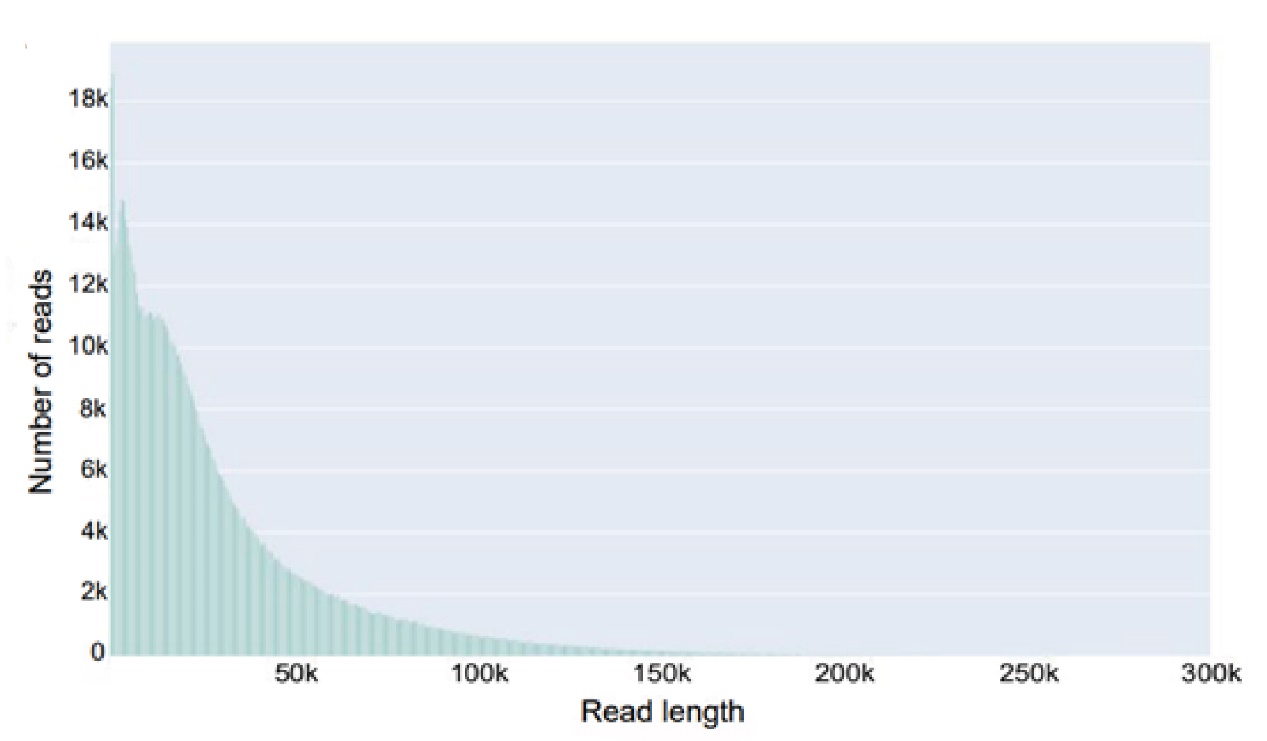


**Supplementary Table S1**| The statistics of Sequencing data of Nanopore ultra-long reads

| Type | No. of bases (Gb) | Average length of reads (bp) | N50 (bp) | Longest reads (bp) | Depth (×) |
| --- | --- | --- | --- | --- | --- |
| NULRs | 29.9 | 30,692 | 52,867 | 520,559 | 79.3× |
